# Diode laser removal of lower lip mucocele: a case report

**DOI:** 10.11604/pamj.2024.48.142.40314

**Published:** 2024-07-31

**Authors:** Youssef Amal, Sarah Tabbai, Hakima Chhoul

**Affiliations:** 1 Faculty of Dental Medicine of Rabat, Mohammed V University in Rabat, Rabat, Morocco

**Keywords:** Mucocele, lower lip, laser diode, midline diastema, case report

## Abstract

Mucocele is a common benign cyst pathology of minor salivary glands of the oral mucosa. The most common location for these lesions is the lower lip. It mainly occurs due to chronic trauma or habit of lip biting or sucking. Such parafunctional habits lead to the alteration of minor salivary glands and mucous accumulation. This case report presents a patient with lower lip mucocele resulting from a lip-sucking habit due to the presence of maxillary central incisor midline diastema. The mucocele removal was performed with a diode laser, and the midline diastema was closed with composite in order to eliminate the parafunctional habit of lip sucking and prevent a possible relapse.

## Introduction

Mucoceles, also known as mucous cysts, are cavities filled with mucus that commonly appear in the oral cavity, involving various areas such as the lip, cheek, tongue, palate, and floor of the mouth. However, it has been observed that a significant majority, ranging from 44% to 79% of mucoceles occur specifically on the lower lip [1,2]. The incidence of mucoceles is generally about 2.5 lesions per 1000 patients, frequently occurring in the second decade of life and affecting both genders in all age groups, with the peak incidence between 10 and 29 years [3]. Mucoceles can develop through two distinct mechanisms: extravasation and retention, with trauma and obstruction of the salivary gland ducts identified as their primary causes [3]. Several methods have been used to remove mucoceles, including conventional surgery, electrosurgery, cryosurgery, micro-marsupialization, marsupialization, and steroid injection. Diode laser was also reported as an alternative to the precedent methods. However, to prevent mucocele recurrence, it´s important to identify and treat the etiology of this lesion [4]. In the present case report, inter-incisal diastema had an important role in developing lip-sucking habits, leading to mucocele formation. Therefore, to prevent relapse, the surgical excision of mucocele using a diode laser was combined with composite diastema closure.

## Patient and observation

**Patient information:** a 13-year-old male presented to the Dentistry Department, the chief complaint was a painless growth on the lower lip, that regresses and enlarges from time to time in the last 2 years. No significant past medical history or relevant past interventions were reported.

**Figure 1:**
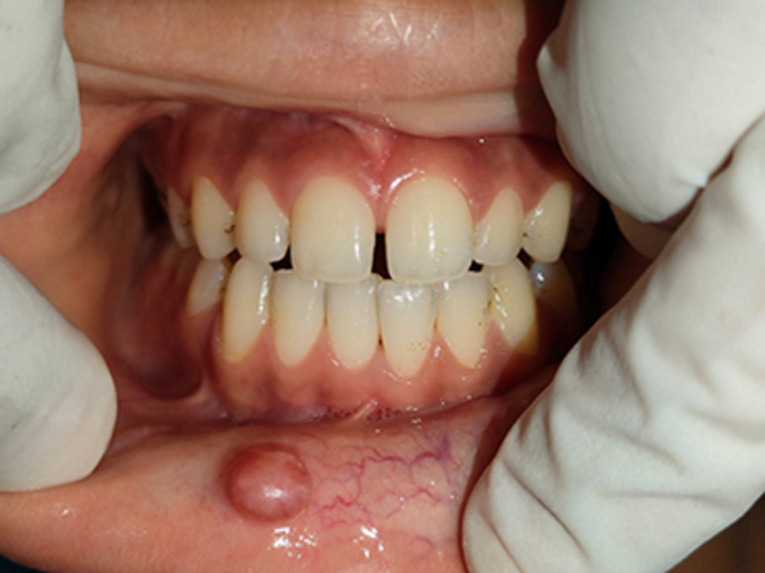
initial clinical presentation of the mucocele in the lower lip and maxillary midline diastema

**Figure 2:**
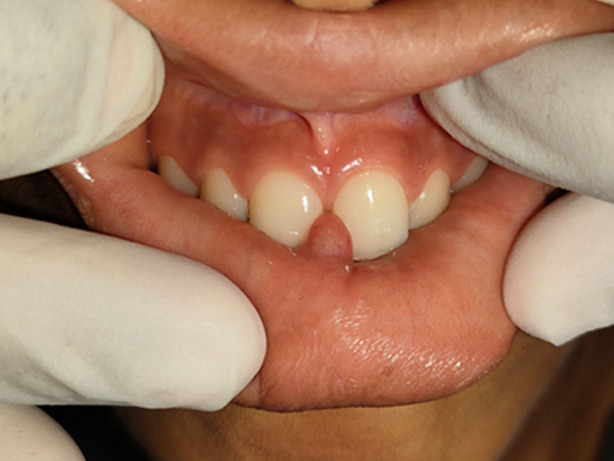
lower lip-sucking habit, demonstrated by drawing the lip into the inter-incisor diastema

**Clinical findings:** the intraoral inspection revealed the presence of an ovoid swelling on the inner aspect of the right side of the lower lip measuring 0.8x0.5x0.4cm, the color of the swelling was the same as that of the adjacent mucosa (Figure 1). On palpation, the swelling was painless, fluctuant in consistency, fluid-filled, and mobile. In addition, the patient presented with an inter-incisor diastema. The child demonstrated a habit of lower lip-sucking by drawing it into the inter-incisor space between the two upper central incisors which may have caused nodule formation (Figure 2).

**Diagnostic assessment:** based on location, progressive growth, and clinical appearance, the diagnosis of a lower lip mucous cyst has been suggested. No diagnostic challenges were reported.

**Figure 3:**
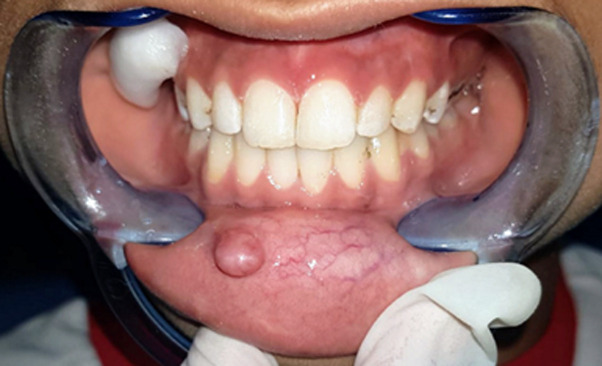
clinical presentation of midline diastema composite closure

**Figure 4:**
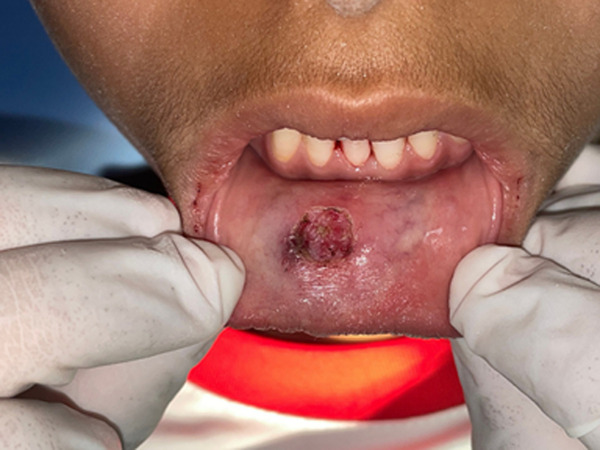
immediate aspect after mucocele removal: bloodless operative site, no suture was done

**Therapeutic intervention:** the treatment consisted of composite diastema closer to help stop the lip-sucking habit (Figure 3), followed by a 940nm diode laser excision. A minimal infiltration of 2% lidocaine with 1: 100,000 epinephrine was performed around the periphery of the lesion, a 3-0 silk suture on the tip of the lesion was used for traction, then the diode laser with a wavelength of 940nm and a 400µm diameter tip was used to excise the lesion in continuous mode at 1.5W. The excision was performed by separating the base of the lesion from the adjacent mucosa, there was no bleeding during or after the procedure and there was no need to suture (Figure 4). The specimen was stored in 10% formalin and sent for histopathological examination. The results showed oral mucosa lined with regular keratinized squamous epithelium. Congested chorion with hemorrhagic changes and pseudocystic cavitation containing a proteinaceous substance interspersed with a granulomatous infiltrate rich in macrophages and bordered by epithelioid elements and rare multinucleated giant cells. Based on these histopathological features, the diagnosis of a mucous extravasation cyst was given. The patient was prescribed analgesics and was advised to stop the habit of lip-sucking to prevent a possible relapse.

**Figure 5:**
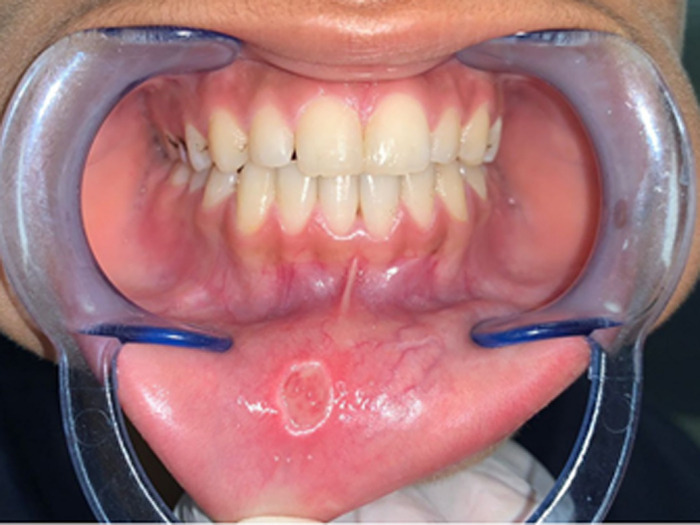
one (1) week follow up

**Figure 6:**
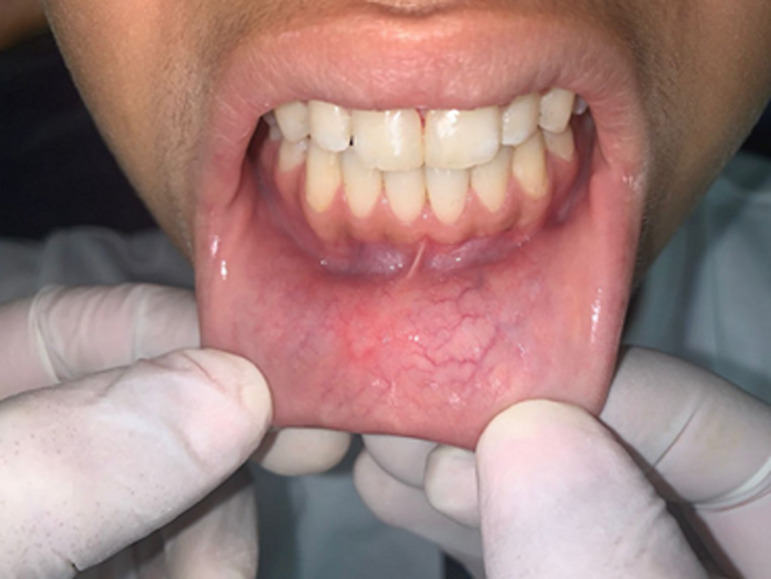
after 3 months follow up

**Follow-up and outcomes:** parents were instructed to come for follow-up visits after 1 week (Figure 5) and 3 months (Figure 6). The wound healed without complications and no occurrence was observed after 3 months of clinical follow-up.

**Patient perspective:** the patient and his parents expressed satisfaction with the treatment outcomes, and the child found that the procedure was painless and did not mention any pain or discomfort during and after the interventions.

**Informed consent:** written informed consent for the case to be published was obtained from the patient and her mother for publication of this case report, including accompanying images

## Discussion

Mucoceles are common benign lesions that often occur in young individuals, they can develop through two mechanisms: extravasation and retention. The extravasation type (84.48%) is more common than the retention type (15.52%) [5]. Extravasation mucoceles develop when fluid leaks from broken ducts or acini of the minor salivary glands into the surrounding submucosal tissue, resulting in stagnant mucous. This leakage is often due to physical trauma or habits of lip-sucking or biting [3]. Extravasation mucoceles progress through three stages of development: i) during the initial phase, mucus leaks out of the excretory duct and spreads throughout the conjunctive tissues where a few leucocytes and histiocytes can be observed; ii) the resorption phase is marked by the appearance of granulomas caused by histocytes, macrophages, and giant multinucleated cells reacting to a foreign body; iii) in the last phase, connective cells create a pseudo-capsule without an epithelium surrounding the mucosa.

Retention mucocele on the other hand develops by a blockage of the salivary gland ducts causing a decrease or absence of glandular secretion [1]. Clinically, mucoceles can be deep in the upper submucosa, where the swellings are well circumscribed and typically covered by normal appearing oral mucosa, or superficial, immediately beneath the mucous membrane, in which case they appear as bluish swellings with thin walls that rupture easily. Oral mucoceles may appear as a fluid-filled vesicle or blister in the superficial mucosa, or fluctuant nodule deep within the connective tissue [5]. Diagnosis is principally based on the clinical presentation of the lesion, therefore, lesion location, history of trauma, rapid appearance, variations in size, bluish color, and consistency are crucial data and should be correctly carried out. Differential diagnosis can be assessed by palpation, lipomas and tumors of minor salivary glands present no fluctuation while cysts, mucoceles, abscesses, and hemangiomas do. Despite this fluctuation, a drained or chronic mucocele with a developed fibrosis would have less fluctuation. The technique of fine needle aspiration biopsy (FNAB) is particularly useful, especially when differentiating between angiomatous lesions is involved [3].

A histopathologic study is crucial to confirm the diagnosis and to ensure that glandular tissue is completely removed. Retention-type mucoceles are characterized by the presence of a cystic cavity that has a clearly defined wall made up of cuboidal cells. This type of mucocele tends to have a lower degree of inflammation. On the other hand, the extravasation type of mucocele is a pseudocyst that does not have an epithelial wall and typically shows the presence of inflammatory cells and granulation tissues. Despite the lack of an epithelial covering around the mucosa, the extravasation-type mucocele is still well-encapsulated [6]. Mucocele has clinical resemblance with many other swellings and ulcerative lesions of oral cavity and hence needs to be differentiated carefully, Neha Bhargava *et al*. attempted to list the probable differential diagnosis of mucocele occurring at a most common site, that is lower lip, along with all the clinical features that helps in their differentiation, the following is an enumeration of the possible conditions: fibroma, lipoma, hemangioma, varix, traumatic neuroma, salivary duct cyst, epidermoid cyst, mucoepidermoid carcinoma, blue nevi, granular cell tumor, lymphangioma, pyogenic granuloma [7].

The conventional approach to managing mucoceles involves surgically removing the adjacent mucosal and glandular tissue down to the muscle layer. However, marsupialization offers a viable alternative, particularly for larger lesions, as it minimizes damage to vital structures. Micro-marsupialization techniques, which aim to drain the mucus and reduce the size of the lesion, involve passing a thick silk suture through the lesion's widest diameter and securing it with a knot. This simple, painless, and minimally invasive method is typically removed after 7-10 days, allowing the mucocele to drain and heal, making it particularly suitable for pediatric patients [8]. Cryosurgery and intralesional steroid injections have also been reported, the main disadvantage of these techniques is the lack of a specimen to be examined microscopically to confirm the diagnosis [9]. Laser diodes have become increasingly popular for managing oral soft tissues due to their excellent absorption by water and hemoglobin, melanin, and collagen chromophores, and poor absorption by dental hard tissues. This feature makes diode lasers a perfect surgical tool for oral soft tissues, as they offer precise cuts without affecting the muscle layer, minimal bleeding, and almost no acute inflammatory reaction. Additionally, the short operation time (3-5 minutes) makes it an ideal treatment option for patients who cannot withstand long procedures, particularly children. There is no need for sutures, and the antibacterial and anti-inflammatory properties of the tool promote wound healing without swelling or infection. However, this type of laser can cause rapid temperature increases in the target tissue, which may lead to adjacent tissue overheating and necrosis. Therefore, careful attention must be given to the time of application and working power to avoid adverse effects [10].

## Conclusion

This case report highlights the significance of identifying the underlying etiology of mucocele development such as trauma, habits of lip sucking and biting, for effective treatment, also indicates that diode laser surgical excision of the lower lip mucocele is an effective treatment method that is well-suited to young patients and to those intolerants of long procedure.

## References

[1] Baurmash HD (2003). Mucoceles and ranulas. J Oral Maxillofac Surg.

[2] Yamasoba T, Tayama N, Syoji M, Fukuta M (1990). Clinicostatistical study of lower lip mucoceles. Head Neck.

[3] Ata-Ali J, Carrillo C, Bonet C, Balaguer J, Peñarrocha Diago M, Peñarrocha M (2010). Oral mucocele: Review of the literature. J Clin Exp Dent.

[4] Besbes A, Elelmi Y, Khanfir F, Belgacem R, Ghedira H (2020). Recurrent oral mucocele management with diode laser. Case Rep Dent.

[5] More CB, Bhavsar K, Varma S, Tailor M (2014). Oral mucocele: a clinical and histopathological study. J Oral Maxillofac Pathol.

[6] Re Cecconi D, Achilli A, Tarozzi M, Lodi G, Demarosi F, Sardella A (2010). Mucoceles of the oral cavity: a large case series (1994-2008) and a literature review. Med Oral Patol Oral Cir Bucal.

[7] Bhargava N, Agarwal P, Sharma N, Agrawal M, Sidiq M, Narain P (2014). An unusual presentation of oral mucocele in infant and its review. Case Rep Dent.

[8] Amaral MBF, De Freitas JB, Mesquita RA (2012). Upgrading of the micro-marsupialisation technique for the management of mucus extravasation or retention phenomena. Int J Oral Maxillofac Surg.

[9] Garg A, Tripathi A, Chowdhry S, Sharma A, Biswas G (2014). Cryosurgery: painless and fearless management of mucocele in young patient. J Clin Diagn Res.

[10] Bagher SM, Sulimany AM, Kaplan M, Loo CY (2018). Treating mucocele in pediatric patients using a diode laser: three case reports. Dent J (Basel).

